# Fasting Blood Glucose-A Missing Variable for GFR-Estimation in Type 1 Diabetes?

**DOI:** 10.1371/journal.pone.0096264

**Published:** 2014-04-29

**Authors:** Petter Bjornstad, R. Brett McQueen, Janet K. Snell-Bergeon, David Cherney, Laura Pyle, Bruce Perkins, Marian Rewers, David M. Maahs

**Affiliations:** 1 Department of Pediatrics, University of Colorado School of Medicine, Aurora, Colorado, United States of America; 2 University of Colorado School of Pharmacy, Aurora, Colorado, United States of America; 3 Barbara Davis Center for Diabetes, University of Colorado School of Medicine, Aurora, Colorado, United States of America; 4 Department of Medicine, Division of Nephrology, Toronto General Hospital, University of Toronto, Ontario, Canada; 5 Department of Biostatistics, University of Colorado Denver, Aurora, Colorado, United States of America; 6 Department of Medicine, Division of Endocrinology, Mount Sinai Hospital, University of Toronto, Ontario, Canada; The University of Manchester, United Kingdom

## Abstract

**Objective:**

Estimation of glomerular filtration rate (eGFR) is one of the current clinical methods for identifying risk for diabetic nephropathy in subjects with type 1 diabetes (T1D). Hyperglycemia is known to influence GFR in T1D and variability in blood glucose at the time of eGFR measurement could introduce bias in eGFR. We hypothesized that simultaneously measured blood glucose would influence eGFR in adults with T1D.

**Methods:**

Longitudinal multivariable mixed-models were employed to investigate the relationships between blood glucose and eGFR by CKD-EPI eGFR_CYSTATIN C_ over 6-years in the Coronary Artery Calcification in Type 1 diabetes (CACTI) study. All subjects with T1D and complete data including blood glucose and cystatin C for at least one of the three visits (n = 616, 554, and 521, respectively) were included in the longitudinal analyses.

**Results:**

In mixed-models adjusting for sex, HbA1c, ACEi/ARB, protein and sodium intake positive associations were observed between simultaneous blood glucose and eGFR_CYSTATIN C_ (β±SE:0.14±0.04 per 10 mg/dL of blood glucose, p<0.0001), and hyperfiltration as a dichotomous outcome (OR: 1.04, 95% CI: 1.01–1.07 per 10 mg/dL of blood glucose, p = 0.02).

**Conclusions:**

In our longitudinal data in subjects with T1D, simultaneous blood glucose has an independent positive effect on eGFR_CYSTATIN C_. The associations between blood glucose and eGFR_CYSTATIN C_ may bias the accurate detection of early diabetic nephropathy, especially in people with longitudinal variability in blood glucose.

## Introduction

Diabetic nephropathy (DN) remains the single most important cause of renal failure in North America, and also one of the major causes of mortality in type 1 diabetes [1], [2]. The American Diabetes Association, National Kidney Foundation and International Society of Nephrology recommend annual screening for albuminuria and also measurement of estimated glomerular filtration rate (eGFR) to identify and monitor DN [3]–[5]. Several factors influencing GFR are well documented and commonly accounted for when employing estimating equations to assess renal function including age, gender, and ethnicity [6]. The effect of hyperglycemia on renal physiology and GFR measurement is well recognized in controlled studies [7], [8], but is not accounted for in GFR estimating equations, which could introduce bias in the 1.5 million patients in the US and 20 million worldwide with type 1 diabetes (T1D) [9]–[11]. Moreover, although eGFR equations have included subjects with T1D [6], only diabetes status was considered and not blood glucose concentrations as potential explanatory variables.

The influence of simultaneous blood glucose on eGFR (using serum creatinine) was suggested by the DCCT-EDIC study which showed a decrease of 4.2 mL/min/1.73 m^2^ in eGFR from baseline to year one among subjects randomized to intensive diabetes control and a sustained lower eGFR during the DCCT; similarly, eGFR decreased 4.8 mL/min/1.73 m^2^ from DCCT close-out to year 1 of EDIC in subjects in the conventional arm after transition to more intensive diabetes management [12]. Furthermore, over 6 ½ years in the DCCT the overall difference in mean daytime blood glucose between intensively and conventionally treated subjects was 76 mg/dL and associated with a 1.7 mL/min/1.73 m^2^ overall increase in eGFR in the conventionally treated group [12], [13]. One hypothesis to explain these findings is the effect of blood glucose control at the time of eGFR measurement.

Compared to creatinine-based equations, we have previously demonstrated that cystatin C has a superior ability to experimentally detect acute changes in GFR induced by hyperglycemia under carefully controlled physiological conditions [14]. However, the role of simultaneously measured glucose on eGFR calculated by using cystatin C equations in people with T1D outside of a controlled laboratory setting is not known. It is important to determine the magnitude of the effect of changes in plasma glucose on eGFR to potentially provide more precise GFR estimates in people with T1D.

Accordingly, our aim was to determine the longitudinal association of simultaneous fasting glucose on eGFR using cystatin C-based equations in the Coronary Artery Calcification in T1D (CACTI) study, a large cohort of adults with T1D, over 6 years to better understand how simultaneously measured blood glucose affects eGFR over time. We hypothesized that eGFR would be positively associated with simultaneously measured blood glucose level. Second, and consistent with previous work we hypothesized that the association between glucose and eGFR would be weaker in patients taking RAAS inhibition (RAASi) than in those without RAASi, since RAASi attenuates the hemodynamic effects of hyperglycemia [15].

## Materials and Methods

### Ethics Statement

The study was approved by the Colorado Multiple Institutional Review Board and all participants provided written informed consent to participate in this study.

### Cohort and Methods

The CACTI Study enrolled subjects 19–56 years old, with and without T1D, who were asymptomatic for cardiovascular disease (CVD) at the baseline visit in 2000–2002 and then were re-examined 3 and 6 years later, as previously described [16]. Subjects with T1D had to have diabetes duration of at least 10 years at enrollment, with the exception of 13 subjects with a shorter duration at baseline who had taken part in a pilot study in 1997–1998 and were grandfathered in to the cohort. Subjects with serum creatinine >2 mg/dL were excluded at baseline, unless they were participants in the pilot study.

Study participants who completed the baseline screening visit were asked to fill out a validated self-administered food-frequency questionnaire, from which we obtained sodium and protein intake [17]. We measured height and weight, and calculated body mass index (BMI) in kg/m^2^. Resting systolic (SBP) and fifth-phase diastolic blood pressure (DBP) were measured three times while the patient was seated, and the second and third measurements were averaged. Hypertension was defined as current anti-hypertensive therapy or untreated hypertension (BP ≥140/90 mmHg) at the time of the study visit. Anti-hypertension medication use was determined by a medication inventory as previously described [16] and use of an ACE inhibitor (ACEi) or an angiotensin receptor blocker (ARB) were combined for these analyses.

After an overnight fast, blood was collected, centrifuged, and separated. Blood glucose was measured using standard enzymatic methods in the laboratory and high performance liquid chromatography was used to measure HbA1c (HPLC, BioRad variant). Serum uric acid (SUA) was measured on stored baseline samples via the Clinical Analyzer utilizing a uricase-based commercial kit. These samples had been thawed twice in the past. The results were reported in milligrams per deciliter (mg/dL). LDL cholesterol (LDL-C) was calculated using the Friedewald formula. Timed overnight urine samples were collected and urine creatinine and albumin were measured (RIA, Diagnostic Products) at all three visits. Urinary albumin excretion rate (AER) was calculated and the results were reported in microgram per min (µg/min). Cystatin C was measured in the University of Colorado Hospital clinical lab using the commercially available Dade-Behring assay following package insert instructions on a BNII or Prospec instrument, as previously described in detail [18]. Due to a systematic shift in the Dade-Behring cystatin C assay over the time period of our study, cystatin C levels were standardized to Visit 3 levels using Deming regression equations as previously described [18]. GFR (ml/min/1.73 m^2^) was determined using the CKD-EPI eGFR_CYSTATIN C_, equation recently published by the CKD-EPI Investigators Group [6].

Categorical analyses were also performed based on eGFR and blood glucose to investigate whether thresholds of hyperfiltration affected these associations. There is no generally accepted definition for hyperfiltration [19]. In our study, we determined sex-specific reference values for hyperfiltration using the upper 90^th^ percentile of eGFR from normal controls of CACTI to define hyperfiltration (eGFR >120 mL/min/1.73 m^2^ reflected approximately the 90^th^ percentile for both male and female controls by CKD-EPI eGFR_CYSTATIN C_).

### Statistical Analysis

Differences between variables were assessed using paired *t*-tests. AER had a non-parametric distribution and were presented as geometric means and natural log-transformed in the multivariable mixed models. To explore the relationships between blood glucose and eGFR as continuous outcome and categorical outcome (hyperfiltration, eGFR > 120 mL/min/1.73 m^2^) at all three visits and how these relationships differ over time, we employed longitudinal linear and logistic mixed-models [20], [21]. These models strengthen the cross-sectional evidence by observing the same associations over repeated time points. To investigate whether the associations between glucose and eGFR and hyperfiltration were independent of confounding variables, we adjusted for sex, sodium intake, protein intake, HbA1c, ACEi/ARB use. We adjusted for HbA1c, as we sought to explore the effects of acute glucose on eGFR rather than long term glycemic control which could confound our results.

The mixed model written in observation-specific form is shown below:


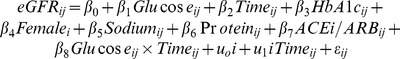
i  =  1, 2, 3, …, n subjects

j  =  1, 2, 3 measurements at each time period or visit

Spot glucose (mg/dL), HbA1c (%), sodium intake, protein intake, and time were treated as continuous variables. Female was coded as dichotomous with male as the reference group. ACEi/ARB was coded as dichotomous with no ACEi/ARB use as the reference group. 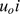
is a random intercept representing the individual intercept deviation. 
is a random slope on time representing the individual slope deviation for time. The random effects have mean 0 and we assumed an unstructured covariance matrix to allow for correlation between the random effects. The interaction term between glucose and time represents the per time period change in spot glucose on eGFR levels (i.e., how does the slope vary across time). The reported association between glucose and eGFR is a marginal effect calculated as 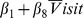, where 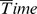
is the mean observed time for participants across all three visits; similar to a pooled estimate across all visits. The marginal effect represents the average difference in eGFR for every 10-unit difference in glucose (e.g., 10 mg/dL for blood glucose), after adjusting for confounding variables. A similar approach was taken for the logistic mixed models using hyperfiltration as a dichotomous outcome, with the following abbreviated specification:
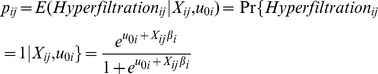



Note that 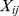
is the same vector of covariates included in the linear mixed model. The model with the best fit statistics included only a random intercept term, 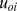, assumed to follow a normal distribution with mean 0 and variance 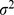. The marginal effect reported is also a function of glucose, and the interacted term between glucose and time. However, the interpretation using mixed effects logistic regression is subject-specific (i.e., conditional on the random effect). That is, for a given patient, there was an x% change in the odds of hyperfiltration for every 10-unit difference in glucose (e.g., 10 mg/dL for blood glucose), after adjusting for confounding variables. Gender and RAASi have both been suggested to influence blood glucose's effect on eGFR, and therefore we present non-stratified models in addition to models stratified by gender and ACEi/ARB therapy. We attempted multiple sensitivity analyses including different model and random effects specifications. We also performed post-hoc sensitivity analyses adjusting for BMI, LDL-C, SUA at baseline, AER and SBP along with the existing variables (sex, HbA1c, ACEi/ARB usage, protein intake and sodium intake) to further assess the relationship between blood glucose and eGFR_CYSTATIN C_ and hyperfiltration. The Akaike Information Criteria (AIC), log-likelihood, and residual plots were used to analyze goodness of fit. All analyses were performed in Stata (Stata Statistical Software: Release 12. College Station, TX: StataCorp LP) using the xtmixed and xtlogit commands. A value of P < 0.05 was considered statistically significant.

## Results

**Figure 1:**
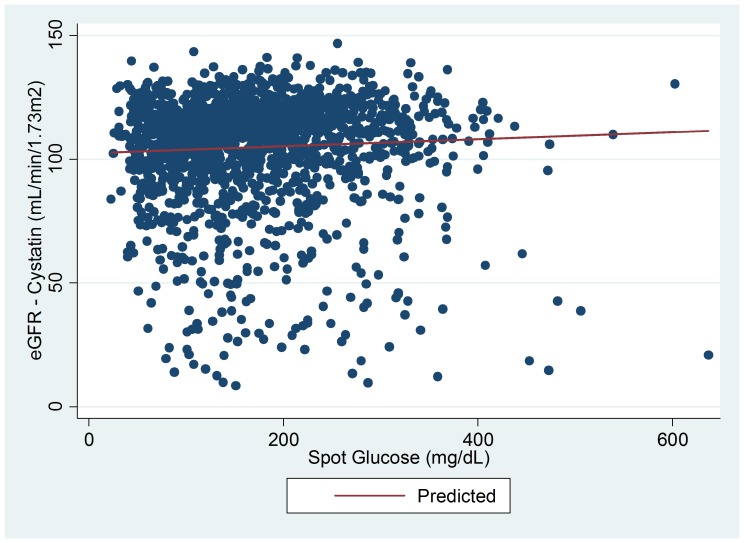
Scatter plot of eGFR vs. spot glucose with fitted values from linear mixed model for the overall population.

The characteristics of subjects with T1D at visit 1, 2 and 3 are summarized in Table 1. In non-stratified mixed-models adjusting for sex, HbA1c, ACEi/ARB usage, protein intake and sodium intake a positive association was observed between simultaneously measured blood glucose and eGFR_CYSTATIN C_ (β±SE: 0.14±0.04, p<0.0001, per 10 mg/dl of blood glucose, **Figure 1**). Stratified by gender the associations between blood glucose and eGFR_CYSTATIN C_ remained significant in both females (β±SE: 0.15±0.05, p = 0.003) and males (β±SE: 0.14±0.06, p = 0.03, Table 2). Moreover, stratified by ACEi/ARB use, subjects without RAASi demonstrated a significant positive association between blood glucose and eGFR_CYSTATIN C_ (β±SE: 0.14±0.05, p = 0.005), in contrast to a non-significant association in those with RAASi (β±SE:0.13±0.07, p = 0.07, Table 2).

**Table 1:** Baseline Subject Characteristics.

	Visit 1, n = 616	Visit 2, n = 544	Visit 3, n = 521	p-value^†^
	Mean±SD/n(%) unless otherwise specified			
**Age (years)**	37±9	39±9	43±9	N/A
**Sex, male/female (%)**	46/54%	46/54%	46/54%	N/A
**Duration of Type 1 Diabetes (years)**	23±9	26±9	29±9	N/A
**BMI (kg/m^2^)**	26.2±4.4	26.5±4.5	26.9±4.8	<0.001
**SUA (mg/dL)**	5.1±1.1	–	–	N/A
**LDL-C (mg/dL)**	100.6±29.2	100.3±27.3	87.5±29.9	<0.001
**Current Smoker (N/%)**	75 (12.5%)	43 (8.7%)	40 (8.1%)	0.01
**Ever a Smoker (N/%)**	119 (19.8%)	109 (22.1%)	151 (30.6%)	<0.01
**Cystatin C (mg/dL)**	0.85±0.39	0.86±0.41	0.85±0.33	<0.01
**eGFR based on cystatin C (mL/min/1.73 m^2^)**	106±22^*^	104±23^*^	102±22^*^	<0.01
**eGFR_CYSTATIN C_ > 120 mL/min/1.73 m^2^ (n/%)**	141 (22.9%)^**^	124 (22.8%)	79 (15.2%)	<0.01
**AER (µg/min)** a	11 (9–12)	11 (9–12)	8 (7–10)	0.04
**Spot Glucose (mg/dL)^b^**	190±95	170±81	156±70	
	(25–637)	(26–539)	(23–375)	<0.01
**HbA1c (%)**	8.0±1.3	7.7±1.2	7.9±1.2	0.2
**Sodium intake**	1997±988	1989±810	2097± 928	0.05
**Protein intake**	86±37	87±35	91±38	0.01
**Hypertension meds (N/%)**	231 (37.7%)	242 (45%)	273 (51%)	<0.01
**ACEi/ARB (N/%)**	210 (34.1%)	190 (40.0%)	248 (45.8%)	<0.01

a Geometric mean and 95% CI. ^b^ Mean ± SD and min-max. * p<0.0001 in all pair-wise comparisons, ** p<0.0001 in all pair-wise comparisons. †p-value testing the mean change over time.

**Table 2:** Multivariable models with eGFR as a continuous and dichotomous outcome (hyperfiltration).

	eGFR Cystatin C		eGFR > 120 mL/min/1.73 m^2^ by Cystatin C	
	β±SE**	p-value	OR, 95% CI***	p-value
**Non-stratified models**				
**Glucose** *	0.14±0.04	<0.0001	1.04 (1.01-1.07)	0.02
**No RAASi (No ACEi/ARB use)**				
**Glucose** *	0.14±0.05	0.005	1.04 (1.00-1.09)	0.03
**RAASi (ACEi/ARB use)**				
**Glucose** *	0.13±0.07	0.07	1.02 (0.96–1.09)	0.43

*Multivariable models adjusted for gender, HbA1c, protein and sodium intake and ACEi/ARB use. **β-coefficient represents the difference in eGFR for every 10-unit increase for glucose (e.g., 10 mg/dL for blood glucose) in the independent variable, and difference for every 1-unit difference for the other variables.

***Odds ratios represent the average odds of hyperfiltration for every 10-unit increase in glucose, and for every 1-unit difference for the other variables.

**Figure 2:**
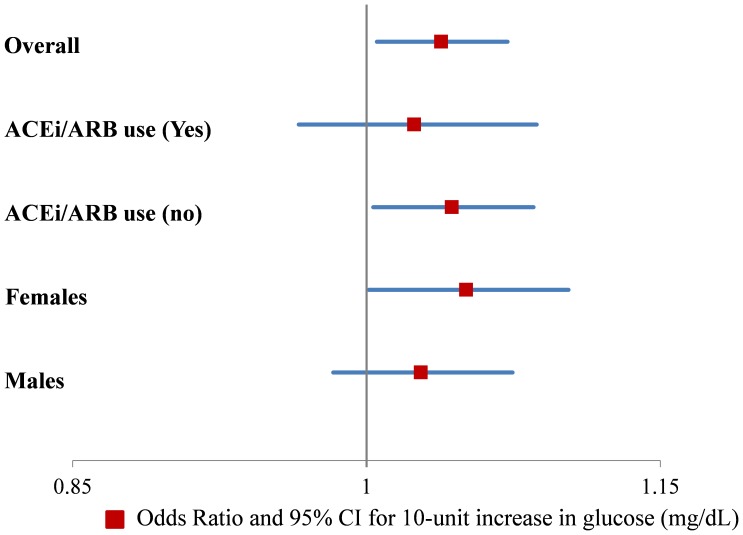
Odds of Hyperfiltration eGFR > 120 mL/min/1.73 m^2^ by Cystatin C for a 10-unit increase in glucose (mg/dL) in multivariable models adjusted for gender, HbA1c, protein inake, sodium intake and ACEi/ARB use for for the overall population and stratified by ACEi/ARB use and gender.

In non-stratified mixed-models with hyperfiltration as a dichotomous outcome, glucose was associated with hyperfiltration (OR = 1.04 [95% CI: 1.01–1.07], p = 0.02) in a fully-adjusted model. This translates to a 4% increase in odds of having hyperfiltration after multivariable-adjustments for every 10 mg/mL higher blood glucose. Stratified by ACEi/ARB therapy, the association between glucose and hyperfiltration was only significant for subjects without RAASi (ACEi/ARB use: OR = 1.02 [95% CI: 0.96–1.09], p = 0.43, no ARBi/ARB use: OR = 1.04 [95% CI: 1.03–1.09], p = 0.03) (**Figure 2**).

To further assess the relationship between blood glucose and eGFR_CYSTATIN C_ and hyperfiltration we performed *post-hoc* sensitivity analyses adjusting for BMI, LDL-C, SUA at baseline, AER and SBP along with the existing variables (sex, HbA1c, ACEi/ARB usage, protein intake and sodium intake). In non-stratified models glucose remains significantly associated with eGFR_ CYSTATIN C_ and hyperfiltration (Table 3). Stratified by ACEi/ARB use, subjects without RAASi demonstrated a significant positive association between blood glucose and eGFR_CYSTATIN C_ and hyperfiltration, in contrast to non-significant associations with eGFR_CYSTATIN C_ and hyperfiltration in those with RAASi (Table 3).

**Table 3:** Post-hoc sensitivity analyses - multivariable models with eGFR as a continuous and dichotomous outcome (hyperfiltration).

	eGFR Cystatin C		eGFR > 120 mL/min/1.73 m^2^ by Cystatin C	
	β±SE**	p-value	OR, 95% CI***	p-value
**Non-stratified models**				
**Glucose** *	0.15±0.04	0.001	1.04 (1.01–1.08)	0.02
**No RAASi (No ACEi/ARB use)**				
**Glucose** *	0.15±0.05	0.003	1.05 (1.01–1.09)	0.02
**RAASi (ACEi/ARB use)**				
**Glucose** *	0.11±0.07	0.15	1.03 (0.96–1.10)	0.45

*Multivariable models adjusted for gender, HbA1c, protein and sodium intake, ACEi/ARB use, LDL-C, BMI, SUA at baseline, LnAER and SBP.

**β-coefficient represents the difference in eGFR for every 10-unit increase for glucose (e.g., 10 mg/dL for blood glucose) in the independent variable, and difference for every 1-unit difference for the other variables.

***Odds ratios represent the average odds of hyperfiltration for every 10-unit increase in glucose, and for every 1-unit difference for the other variables.

## Discussion

Our major findings in this descriptive study were that simultaneously measured blood glucose was positively associated with eGFR and with hyperfiltration calculated by eGFR_CYSTATIN C_ in subjects with T1D after multivariable adjustments, which is consistent with experimental data [14] and with our hypothesis. The rationale for our study was twofold: 1) simultaneous hyperglycemia is known to affect GFR under experimental conditions [14], [22]–[24]; and 2) while simultaneous hyperglycemia may increase GFR under controlled settings, the association of blood glucose variability with cystatin C based eGFR measurements is uncertain in patients with T1D in clinical studies or clinical care settings.

This question is clinically important as eGFR and microalbuminuria are the only two methods recommended by American Diabetes Association, National Kidney Foundation and International Society of Nephrology to screen for and monitor progression of DN in the clinical setting [3]–[5]. As microalbuminuria does not necessarily lead to macroalbuminuria, and in fact may regress spontaneously [25], [26], estimation of GFR has an important role in risk stratification. Accordingly, clinicians rely upon calculations of eGFR to identify early DN (e.g. rapid GFR decline [annual loss >3mL/min/1.73 m^2^]) and risk-stratify subjects with T1D [27]–[29]. Furthermore, a recent report by Krolewski et al. shows that rapid GFR decline occurs prior to the onset of microalbuminuria [30] supporting its role in early risk stratification. Recent data from the DCCT-EDIC study suggested that blood glucose at the time of eGFR measurement may bias results, as subjects randomized to intensive diabetes control displayed a lower eGFR at one year in the DCCT and also in EDIC among those in the DCCT conventional arm who then intensified their blood glucose control [12].

The etiology of increased GFR in the setting of elevated blood glucose in subjects with T1D is incompletely understood, but has been attributed to the effect of hyperglycemia on RAAS. Miller et al. demonstrated that hyperfiltration responses to clamped hyperglycemia are related to intrarenal RAAS activation [15], [31]. Moreover, Cherney et al. recently showed that RAAS blockade by aliskiren (a direct renin inhibitor) blunts the increased GFR as measured by inulin clearance provoked by hyperglycemia [32]. These findings are also consistent with our data, where we did not observe a significant association between glucose and eGFR_CYSTATIN C_ in subjects with RAAS inhibition. Furthermore, hyperglycemia has been proposed to increase proximal tubular glucose delivery causing a maladaptive increase in glucose reabsorption along with sodium via sodium-glucose cotransporter 2 (SGLT-2) in the proximal tube. Consequently – distal sodium chloride delivery to the macula densa is decreased. This decrease is perceived as low effective circulating volume by the juxtaglomerular apparatus, which causes vasodilation of the afferent renal arteriole and an increased GFR [33]. There is to our knowledge no current evidence of a direct effect of glucose on cystatin C, suggesting the positive relationship between blood glucose and eGFR_CYSTATIN C_ is likely reflective of changes in GFR.

Previous experimental studies to date suggest a positive relationship between fasting blood glucose and measured GFR, an association which is consistent with the relationship between simultaneously measured blood glucose and eGFR_CYSTATIN C_ in our study. The CKD-EPI equations estimate GFR, but have increased bias and less precision when GFR is >60 mL/min/1.73 m^2^ [6]. Previous experimental work has consistently demonstrated that serum creatinine-based methods cannot detect acute changes in GFR [14], [34]. The literature is less consistent with regards to cystatin C [14]. Cherney et al reported that GFR measured by inulin clearance results in a GFR difference of 15–18 mL/min/1.73 m^2^ when blood glucose is clamped at 4–6 mmol/L vs. 9–11 mmol/L in adult patients with T1D [14]. Moreover, they demonstrated a strong agreement between eGFR_CYSTATIN C_ and GFR_INULIN_ in detecting increases in GFR provoked by clamped hyperglycemia [14] in subjects with T1D, but this relationship was not evident using eGFR_CREATININE_. In contrast, Melsom et al did not demonstrate statistical agreement between eGFR_CYSTATIN C_ and GFR_IOHEXOL_ in detecting GFR changes, however this was in non-diabetic individuals with impaired fasting glucose in whom there is less blood glucose variability than in people with T1D [34].

The utility of cystatin C as a marker of renal function versus creatinine based estimates is controversial, as reviewed elsewhere [35]. Cystatin C was used in this study instead of creatinine due to its superior operating characteristics as a measure of renal function changes in response to ambient glycemia. eGFR_CYSTATIN C_ is considered to be less biased by age and weight compared to creatinine-based measurements [27]. It is also recognized that blood glucose concentrations above 300 mg/dL may cause an overestimation of serum creatinine levels (and consequently an underestimation of eGFR_CREATININE_) [36], [37]. Moreover, eGFR_CYSTATIN C_ appears to predict micro- and macrovascular complications in subjects with T1D better than eGFR_CREATININE_ [29], [38], [39]. Skupien et al. also recently demonstrated that GFR staging with eGFR_CYSTATIN C_ is superior for predicting ESRD and mortality than staging by the combined eGFR creatinine and cystatin C equation, which they hypothesized might suggest that some determinant of serum creatinine counters the predictive effect of serum cystatin C [40]. Finally, Shlipak et al. recently demonstrated that the use of eGFR_CYSTATIN C_ compared to eGFR_CREATININE_ strengthens the association between eGFR and risk of death and end-stage renal disease in 11 diverse general-population studies [41].

An important question derived from our data is whether the statistically significant associations observed between simultaneously measured blood glucose and eGFR_CYSTATIN C_ is clinically significant and warrants systematic adjustment for blood glucose when employing these equations to estimate GFR in subjects with T1D. In fully adjusted models, an increase in simultaneously measured blood glucose of 10 mg/dL would be associated with an eGFR_CYSTATIN C_ increase of 0.14 mL/min/1.73 m^2^, or 1.4 mL/min/1.73 m^2^ for an increase of 100 mg/dL. Glycemic excursions greater than 100 mg/dL are not uncommon in people with type 1 diabetes. With rapid GFR decline defined as an annual loss >3 mL/min/1.73 m^2^ or >3.3% [25], [28], such excursions could significantly bias the detection of early DN. For that reason, adding blood glucose to eGFR estimation in people with T1D might improve intra-individual precision over time, which could ultimately improve screening, detection, and prevention of early changes in the GFR [42]. Early identification of subjects at risk of DN is imperative to prevent early morbidity and mortality [2].

Experimentally, the association between blood glucose and measured GFR has been well described [14], [34], [43], but this is to our knowledge the first study exploring the longitudinal relationships between simultaneously measured fasting blood glucose and eGFR, measured by CKD-EPI cystatin C, in a large clinical cohort of subjects with T1D. Nevertheless, there are important limitations to the present study worth mentioning, including the observational design. Moreover, experimental studies can control blood glucose prior to measuring GFR, whereas we had to rely on simultaneously measured fasting blood glucose to represent blood glucose control in the period prior to eGFR measurement, which is known to affect renal hemodynamics. The blood glucose samples in our study were drawn from fasting subjects, and therefore the variability in blood glucose would likely be greater in non-fasting samples. Another limitation was the use of cystatin C based equation rather than direct measurements of GFR, which would have been too cumbersome for use in a large-scale clinical study like CACTI. Without the comparison of a direct measure of GFR we were unable to create a cystatin C based eGFR algorithms adjusting for glucose. More importantly our main goal was to elucidate the effects of simultaneously measured blood glucose on the cystatin C-based estimating equation that are commonly used in research in people with T1D. Although we adjusted for a variety of important confounding variables, we cannot rule out the presence of other risk factors that may have biased the present analyses. However, the purpose of our analyses was not to test the independence relationship between blood glucose and GFR as this is well recognized, but rather elucidate the magnitude of the relationship between blood glucose and eGFR_CYSTATIN C_ outside of the laboratory setting.

In summary, we report a positive independent effect of simultaneously measured blood glucose on eGFR and hyperfiltration by eGFR_CYSTATIN C_ in subjects with T1D over time. The magnitude of the β-coefficient between glucose and eGFR_CYSTATIN C_ may be considered of modest clinical significance. In contrast, in subjects with T1D who experience significant glycemic excursions, the magnitude of the associations may bias the detection of early DN. Further study with measured GFR is required to determine whether systematic adjustments for simultaneously measured glucose in GFR equations for adults with T1D are warranted.
